# Cognitive Facilitation Following Intentional Odor Exposure

**DOI:** 10.3390/s110505469

**Published:** 2011-05-19

**Authors:** Andrew J. Johnson

**Affiliations:** Department of Psychology, Coventry University, UK; E-Mail: Andrew.Johnson@Coventry.ac.uk; Tel.: +44(0)24-7688-8654

**Keywords:** odors, cognitive facilitation, memory, alertness, essential oils

## Abstract

This paper reviews evidence that, in addition to incidental olfactory pollutants, intentional odor delivery can impact cognitive operations both positively and negatively. Evidence for cognitive facilitation/interference is reviewed alongside four potential explanations for odor-induced effects. It is concluded that the pharmacological properties of odors can induce changes in cognition. However, these effects can be accentuated/attenuated by the shift in mood following odor exposure, expectancy of cognitive effects, and cues to behavior via the contextual association with the odor. It is proposed that greater consideration is required in the intentional utilization of odors within both industrial and private locations, since differential effects are observed for odors with positive hedonic qualities.

## 1. Introduction

Odors have been shown to impact human performance across a range of contexts. Indeed, the focus of the current special issue reflects incidental industrial/environmental odor exposure, e.g., [1,2]. This issue has, for example, shown that the inter-relation between malodors is not a simple additive/synergistic process [1,2]. These findings can, therefore, guide how malodors can be masked within urban areas. Since (indirect) instrumental measures of odor detection have been shown to correlate with direct (olfactometry) measures of odor threshold [3], it is important to consider how the instrumental measures of odor presence within populated areas may be affecting human cognitive performance. For example, Rotton [4] found that exposure to a malodor (ethanethiol) negatively affected performance on a complex task (proofreading) but not a simple mental arithmetic task. Rotton [4] argued that malodors exert analogous detrimental effects on attention to that of other sensory pollutants such as noise. The malodor used by Rotton [4] was shown to have negative effects on well-being, further illustrating the noxious characteristics of the stimuli. However, a number of other studies have shown that positively rated odors can exert negative (as well as positive) effects on cognition, e.g., [5–9]. These findings illustrate that it is not a simple relationship between odor valence and cognitive performance, but that positively rated odors can have both positive and negative effects on cognition. Such observations are of importance with the increased use of commercial odorants in both personal and professional environments. Indeed, one must not only consider odors epiphenomenal to industrial operations, but also the intentional utilization of ‘pleasant’ odors in commercial settings. In order to maximize the effectiveness of these odors one must isolate the effects of specific odors and understand the mechanism(s) underpinning such effects.

Essential oils and other commercially available scents have, for example, been shown to positively affect memory, e.g., [5,6], vigilance, e.g., [7,10], pain perception, e.g., [11,12], self-perception/confidence [13], consumer decision making, e.g., [14], and alertness, e.g., [8]. Jellinek [15] identified four potential mechanisms that might explain the odor-induced cognitive facilitation. The first concerns an odor-specific pharmacological mechanism, wherein volatile compounds enter the bloodstream following inhalation and impact neural activity. Such an account predicts odor-specific effects (potentially independent from odor valence). The second explanation is an epiphenomenal hedonically-driven mechanism, wherein effects on cognition are secondary to the increase in mood following odor exposure. Such an account predicts that similar valence odors would produce analogous effects on cognition. The third explanation is that odor effects are purely psychological, in that a prior belief/expectancy pertaining to the qualities of the odor underpins any benefits. Under such an account one might predict that an odor should only impact cognition when: (1) the participant is aware of the odor’s presence, and (2) the participant possesses a belief that the odor should induce specific effects. The final explanation is a contextual/associative account, such that odors have specific effects because their presence has been associated with a particular stimulus/mood/behavior. From a memory perspective, this account would predict that if a participant learnt material in the presence of a specific odor, re-presentation of that odor would operate as a subsequent cue to recall for the learnt material (see [16] for a review of context-dependent memory effects). The current paper will review the evidence that odors presented intentionally (rather than arising following industrial/urban activity) can facilitate/decrement cognitive performance. This concerns odors that are used deliberately because it is thought that particular odorants may benefit mood/cognition (*i.e*., aromatherapy) or, more generally, simply because it is thought that such odors might improve the ambience of an environment. This review will be structured in terms of Jellinek’s [15] four potential mechanisms.

## 2. Pharmacological Facilitation

Pharmacological properties of the odor are one mechanism by which odor exposure may facilitate cognition [15]. Odor-specific compounds may stimulate the olfactory nerve which has close association with the limbic system (an area of the brain associated with cognitive functioning). Furthermore, volatile compounds of the inhaled odor may enter the bloodstream via the nasal mucosa (e.g., animal studies have reported traces of the compound in the blood stream after odor exposure, [17]) and thus impact neural activity following neurological delivery. However, there are two clear limitations to this proposition: first, as yet, human studies have not reported traces in the bloodstream following odor inhalation [18]. Second, the amount of active compounds internalized via inhalation is greatly reduced compared to more direct methods of consumption such as ingestion [18]; as a consequence, any pharmacological effects should be greatly attenuated.

### 2.1. Odor Compound Effects without Inhalation (Anxiety)

Notwithstanding, the above limitation of dose via inhalation, one must first establish whether the compounds are psychoactive and affect cognition without the olfactory process. One possible method through which the pharmacological properties of odors can be examined is via a non-inhalation route of administration. If essential oils can be delivered without knowledge/expectancy of that substance and without the enjoyable experience of pleasant odor exposure, one can examine the chemical properties of the substance.

Lavender inhalation (relative to a non-odor control) has been shown to reduce salivary chromogranin A (a marker of stress) following exposure to a stress-inducing arithmetic task [19]; for other anxiolytic effects see [20,21]. However, rather than pharmacologically-driven, such effects may be secondary to mood elevation from olfactory stimulation or underpinned via expectancy. These issues were addressed in a 6-week double-blind randomized control study comparing the anxiolytic effects of lavender oil capsules and benzodiazepine on patients with Generalized Anxiety Disorder [22]. The study reported no significant differences between lavender oil and benzodiazepine on self-rated measures of anxiety and worry, indicating that lavender’s anxiolytic properties are pharmacologically analogous to anti-anxiety drugs.

Fundamental differences do, however, exist in respect to the effects of dose dependency following inhalation and ingestion. The aforementioned pharmacological effects of lavender [22] appear to require chronic administration, as single dose non-inhalation studies report greatly reduced effects [23,24]. For example, in a sub-clinical study, Bradley *et al*. [24] administered lavender oil capsules (or sunflower oil controls) prior to watching a neutral and anxiety-inducing film clip. Higher doses of lavender (200 μL rather than 100 μL) were found to induce a greater reduction in state anxiety, reduce galvanic skin responses and heart rate, and increase heart rate variability. However, this was only found following the neutral film. This finding has two interesting implications. First, the profound anxiolytic effects of lavender capsules [22] require chronic administration, as the present study found that lavender did not impact participants during the anxiety-inducing film clip. Second, dose dependent effects strengthen the proposal that effects are pharmacological, *i.e*., the more one receives the stronger the effect. Similar diluted effects were reported by Heuberger *et al*. [23] who administrated linalool (a constituent of lavender) or a control peanut oil via massage into the skin (pure air was administered via a mask to prevent inhalation). No subjective effects on well-being were reported, although some physiological deactivation following linalool exposure (decrease in systolic blood pressure and small decrease in skin temperature) suggests some relaxing properties.

The above findings suggest that when administration of the substance is not inhaled, lavender has some pharmacological anxiolytic effect irrespective of the olfactory process. This is further supported by dose dependent effects [24]. However, these effects are greatly attenuated following acute administration. Indeed, profound physiological reductions in stress following lavender inhalation [19] are inconsistent with the limited effects on relaxation when lavender is administrated via means other than inhalation [24]. This is especially important considering that oral administration would provide a far more effective/abundant delivery of the compound than inhalation. This suggests that although lavender has pharmacological effects, inhalation provides additive effects to the basic pharmacological properties.

### 2.2. Evaluation of Pharmacological Account

As noted above, there is some evidence that the pharmacological properties of odors can affect humans in the absence of both the olfactory process and knowledge of ingestion. Indeed, physiological, mood, and cognitive effects have been reported for odors presented at concentrations below the detection threshold [25,26]; suggesting that the conscious experience of odor perception is not required to induce effects on both mood and cognition. With no awareness, it seems unlikely that effects can be driven by expectancy or semantic/contextual association. Such pharmacological effects are however, complicated further following the effects of odor mixing [1,2].

However, it should be noted that even if ingestion of olfactory compounds has psychoactive properties, it does not demonstrate, *per se*, that these compounds are inducing the effects following inhalation. Indeed, in terms of a purely pharmacological explanation for post-inhalation effects, Herz [18] questions whether the speed at which effects are observed are consistent with the 20-minutes required for the compounds to enter the bloodstream and cross the blood-brain barrier. As described above, the oral administration of essential oils demonstrates some pharmacological effects [22–24]; however, the accentuation of such effects when inhaled suggests additive effects of the olfactory process (e.g., mood elevation, expectancy *etc*.). Indeed, Herz [18] notes that it is yet to be examined “whether molecules that smell the same but are chemically different produce the same effects (or not)” (p. 273); such a test would differentiate between the psychological and pharmacological properties of the odor.

Finally, the above observation of cognitive/mood effects without conscious awareness or the experience of inhalation does not directly counter the argument that any effects are epiphenomenal to mood change. One might argue, therefore, that the odor compounds (whether inhaled or orally ingested) affect mood and that this change in mood impacts cognitive performance. To counter such an explanation, one requires evidence of odor specificity, wherein different odors are hedonically similar but differentially affect cognition.

## 3. Facilitation Secondary to Mood Elevation

An alternative account to the pharmacological hypothesis is that improvements/decrements to cognition may be secondary to changes in mood [15]. Elevation in positive mood is linked with improvements in cognition (e.g., [27]); therefore, mood (induced via odor pleasantness) may underpin cognitive facilitation, rather than pharmacological properties of the odor *per se*. Under such an account one might predict that odors with similar hedonic properties would produce similar effects on cognition.

The link between odor presentation and emotion is well established. For example, neurologically, dysfunction in areas of the brain associated with emotion (*i.e*., the limbic system) is related to odor sensitivity [28]. Furthermore, odor-evoked memories have been shown to activate the amygdala, indicating an association with the emotional component of the memory [29]. Behaviorally, positively/negatively valence odors induce emotionally congruent facial expressions in children [30,31], whilst positive odors have been shown to elevate the pitch of spoken language [32] (wherein spoken pitch is linked to emotional state, [33]) and increase the emotional content of memory reports [34]. In addition, the startle response, an emotionally-driven alarm reflex, is influenced by odor exposure [35], further indicating inter-relation.

More explicitly, as aforementioned, a number of studies have shown that odor exposure can impact self-rating mood measures, e.g., [4–6,11,20,36–39]. Physiological responses have provided some support for this shift in mood. Brauchli *et al*. [39] reported that presentation of a negative odor (valeric acid) increased both heart rate and skin concordance; whilst presentation of a positive odor (phenyl ethyl alcohol) decreased both measures (effects with EEG were less clear). Nevertheless, evidence that odor exposure can influence mood does not, *per se*, show that the elevation in mood is driving cognitive improvement. Indeed, it is arguably very difficult to empirically test whether the shift in mood is the causal factor in cognitive facilitation. Knasko [36] has proposed that personality factors (specifically locus of control) may be fundamental in determining whether odors affect the mood of participants. Moreover, past experience of odors can change the neural networks within the olfactory bulb; this can produce profound differences in both the experience and perception of odors (see [41] for review). Consequently, a pilot study identifying positive and negative valence odors (e.g., [10]) may be insufficient in guaranteeing the desired affective responses. In order to fully examine the mood elevation hypothesis it is therefore necessary to obtain mood ratings by the participants to establish (1) if the odors have produced the desired affective response and (2) whether there is a mismatch between cognitive facilitation and mood elevation. Such an investigation was conducted by Heuberger *et al*. [42] and revealed that both self-rated and physiological effects were related to the subjective hedonic evaluations of the odors (see also [12,43]).

### 3.1. Comparisons Following the Induction of Positive Affect

Notwithstanding the aforementioned concerns, Baron and colleagues [44–46] have conducted a number of studies examining whether positive odors have similar effects on cognition to other stimuli considered to elevate mood (*i.e*., receipt of a gift). Employing an anagram task, Baron and Thomley [44] found that more words were found in both the positive odor (lemon and floral) and gift conditions, compared to the control condition. Consistent with predictions of the mood elevation hypothesis, the improvement in cognitive performance was mirrored by an elevation in positive mood for both the odor and gift conditions (compared to the control condition). These effects were broadly replicated employing a word construction task [45]. Both studies support the proposition that a rise in positive affect may underpin cognitive improvement.

However, in a similar design Baron and Kalsher [46] examined the role of positive stimuli (*i.e*., a positive odor and receipt of a small gift) on a driving simulator task. Four conditions were employed whereby participants (1) were given a gift, (2) received a positive odor, (3) received both odor and gift, and (4) received neither gift nor odor (control). Both the positive odor and the gift interventions were found to improve task performance (but not when combined). The authors claimed that the beneficial effects of the odor and gift were due to analogous mechanisms, whereby an increase in positive affect facilitates task performance. However, a clear limitation to this explanation relates to the absence of any self-rated mood effects across the conditions. This dissociation between behavioral and self-rated mood data is also demonstrated by Warm *et al*. [7] where both muguet and peppermint improved performance on the vigilance task but neither odor affected subjective ratings. These findings can be interpreted in two ways. First, mood elevation occurred (and consequently improved performance) in both Baron and Kalsher [46] and Warm *et al*. [7], however in both studies participants were unable to introspect on the increment. This explanation is unsatisfactory as both non-falsifiable and atheoretical: *i.e*., why might participants be unable to introspect on the mood change in Baron and Kalsher [46] but be aware of the elevation in Baron and Thomley [44]? The second explanation is that although positive affect may influence cognitive performance, odors can influence performance in the absence of affective change. Indeed, this interpretation is consistent with Moss and colleagues [6] wherein improvements in memory following peppermint exposure where not mirrored by changes to positive affect (*i.e*., contentedness and calmness).

### 3.2. Pain Perception and Mood

Experimental manipulations of pain using olfactory stimuli are thought to be mediated via odor-induced changes in mood, e.g., [12], with ratings of unpleasantness the best predictor of increases in pain intensity [12,47]. Villemure *et al*. [12] found that experimental heat pain was rated as greater when unpleasant odors, compared to pleasant odors were presented. In a further study it was hypothesized that activation of the sympathetic nervous system via odor exposure may be underpinning the changes in pain perception [47]; that is, unpleasant odors increase activation of the sympathetic nervous system, this induces stress and accentuates the perception of pain. In support of this proposition, galvanic skin responses (GSR) were found to be increased following presentation of an unpleasant odor compared to a pleasant odor [47]. These changes were argued to be due to the hedonic characteristics of the odor.

However, it should be noted that the effects of odors on pain perception have been unreliable. Marchand and Arsenault [11] presented participants with positive, negative, and neutral odors whilst they placed their hand in hot water and rated the thermal stimulation (pain) in 15 s intervals. Self rated mood measures correlated strongly with the pleasantness rating of the odor (r = 0.56), suggesting that the odors were inducing mood changes. The odors had no effect on pain perception in male participants, whereas in female participants pain perception was significantly lower with the pleasant odor compared to the unpleasant odor. However, since mood increases were broadly equivalent across males and females, it seems unlikely that mood elevation was the mechanism that enabled female participants to tolerate more pain in the pleasant odor condition. Furthermore, in a similar design Martin [48] employed a cold-presser task to manipulate pain and despite utilizing odors previously rated as pleasant (lemon) and unpleasant (machine oil), odors did not reduce pain perception relative to the no odor control condition. However, Martin [48] did not measure mood effects following odor exposure; therefore it is unclear to what extent mood elevation was separate from perceptions of pain.

### 3.3. Odor Specificity

If odor effects are secondary to an odor-induced elevation in mood, one might predict that similar valance odors produce qualitatively equivalent effects on cognition and mood. It should be noted that the observation of odor specificity does not, *per se*, demonstrate that the effects are psychopharmacological (indeed, odor specificity may be induced by the expectancy or associative effects outlined by Jellinek [15]); however, if mood elevation alone is the mechanism underpinning facilitation, effects should be uniquely related to the perceived pleasantness of the odors.

#### 3.3.1. Memory

In contrast to the mood hypothesis, odors have been shown to produce differential effects on memory despite the odors having similar hedonic qualities. In a series of studies, Moss and colleagues examined the effect of five odors on performance of the Cognitive Drug Research (CDR) assessment battery [5,6,9]. This battery of tests provides differing measurements of memory; including speed of memory, quality of memory, working memory, and secondary memory. Moss *et al*. [5] compared the effects of lavender, rosemary, and no odor across three groups. It is important to note that for an odor to have facilitative effects on memory, performance must be significantly greater than the control no odor condition. This was found only for secondary memory (an aggregate of performance across long-term memory tasks), wherein rosemary was significantly greater than both lavender and control. Although rosemary was superior to lavender for measures of working memory and quality of memory (an aggregate of memory accuracy across the tasks), performance was not significantly greater than the non-odor (control) condition. This suggests that the significant difference may be underpinned via the damaging effect of lavender on memory. Furthermore, they found that speed of memory was significantly slower in both odor conditions compared to the no odor group [5]. This suggests that the benefit observed for rosemary in secondary memory was not a result of faster processing time. The findings therefore indicate that rosemary benefits long-term retention of items but not the manipulation of items or speed at which items can be accessed from memory. The null effects of lavender on memory are consistent with Ludvigson and Rottman [49] who found no effect of both lavender and cloves on memory. Both Moss *et al*. [5] and Ludvigson and Rottman [49] employed prolonged odor exposure, therefore it is unclear as to what extent the absence of lavender effects were due to habituation. However, it is unclear why the effects of lavender should be sensitive to habituation but rosemary not.

In a very similar design, Moss *et al*. [6] compared exposure to peppermint odor, ylang-ylang odor, and a no odor control group on performance of the CDR. Limited effects on memory were again observed. Memory quality (an aggregate of all the memory tasks: word recall, spatial memory, numeric working memory, delayed word recall, and delayed picture recognition) for the peppermint group was found to be significantly superior than both the ylang-ylang and no odor groups. However, although peppermint was significantly superior to ylang-ylang for both working memory and secondary memory, peppermint was not significantly better than the control group for these measures. This indicates that peppermint does not, *per se*, facilitate secondary and working memory. Speed of memory was reduced in the ylang-ylang group relative to controls, demonstrating analogous effects to both rosemary and lavender [5]. The differential effects across the Moss *et al*. [5,6] studies indicate that the effects of odors may be substance specific. For example, in Moss *et al*. [6] peppermint was shown to facilitate memory quality but did not slow speed of memory, whereas in Moss *et al*. [5], rosemary improved secondary memory but slowed memory performance. This suggests that there is not a simple detrimental relationship between odor exposure and speed of processing. Nor can the facilitative effects of rosemary and peppermint be explained via analogous changes in positive mood. Specifically, rosemary increased contentedness (relative to controls and ylang-ylang) and had no effect on calmness [5]; in contrast, peppermint had no effect on contentedness and calmness did not differ from the no-odor control [6]. Furthermore, although the rosemary group reported significantly higher self-rated alertness (relative to controls), this was not found with the peppermint group. This illustrates that the aforementioned effects cannot simply be explained by a general odor-induced elevation of alertness.

The substance-specific effects reported by Moss *et al*. [5,6] do suggest that the effects of odors are not underpinned via more general effects of odors exposure. Moreover, effects of memory were found independently of increases in positive affective state, e.g., peppermint [6]. Such findings are inconsistent with the mood account and appear to relate to specific odors (driven pharmacologically or semantically).

#### 3.3.2. Alertness/Vigilance

Across a range of studies, odor exposure has been found to affect alertness and vigilance, e.g., [7,8]; for indirect facilitative effects see [50]. A relationship has been found between odor identification ability and resistance of vigilance to sleep deprivation [51]. Such a finding, although not causal, implicates frontal lobe activation in both tasks (for odor projections see [52]).

Similar to the memory arguments above, differential effects for positively valence odors would suggest that mood change is not the mechanism underpinning cognitive effects (and that these effects may be specific to the odor). Such a finding has been reported for a visual attention/vigilance paradigm, with odors similar in both hedonic rating and intensity (phenyl ethyl alcohol and allyl isothiocynanate) producing differential effects [53,54]. In this study the trigeminal odor disrupted performance in the distraction condition and the non-trigeminal odor attenuated the effects of the distracter. One might therefore argue that the effects were underpinned by the trigeminal properties of the odor rather than mood/intensity.

A profound example of two positively hedonic odors that have differential effects on alertness and vigilance is that of peppermint and lavender. In respect to peppermint, a number of studies have examined the effect on alertness and vigilance due to a common association with stimulating properties [55]. For example, Warm *et al*. [7] found that peppermint facilitated the performance of a vigilance task that necessitated the detection of changes in a visual form. Furthermore, physiological assessment reflects the subjective elevation of alertness following peppermint inhalation. For example, Norrish and Dwyer [8] examined daytime sleepiness (a correlate with self-rated alertness [56]) using the Pupillary Sleepiness Test (PST). The increase in pupillary unrest (a measure of sleepiness) following 11-minutes within a darkened laboratory was found to be attenuated in the peppermint odor condition compared to the no-odor control. That is, peppermint was shown to reduce the degree to which an individual became sleepy. In addition, peppermint has been shown to affect electrophysiological activity during sleep [57].

In contrast to the odor specificity above, peppermint and cinnamon have both been found to increase self-rated alertness in a driving simulator task [58]. Analogous alertness findings for cinnamon and peppermint indicate that effects may be more general and not (pharmacologically) odor-specific. However, importantly for odor-specificity (which may be underpinned via psychopharmacological properties of the odor), reductions in perceived anxiety and fatigue were unique to peppermint [58].

Notwithstanding the aforementioned physiological effects, it should be noted that the alerting properties of peppermint can be unreliable. For example, following completion of the CDR battery of tests, although Moss *et al*. [6] reported a significantly higher alertness for participants exposed to peppermint compared to those exposed to ylang-ylang; however, peppermint was not significantly greater than the non-odor (control) condition. This suggests that peppermint does not increase alertness *per se*. Furthermore, in a study by Gould and Martin [10] the participants detected visual signals in a vigilance task; no effect of peppermint was found (see also [59]). The Gould and Martin [10] null finding is curious considering that the task had similar demands to that of Warm *et al*. [7] where beneficial effects were reported. One possible explanation for these conflicting findings may relate to habituation. In Warm *et al*. [7] participants were presented with 30 s bursts of the odorants every 5-min via a mask; whereas in Gould and Martin [10] the odor was present throughout the 20-min vigilance task (this is similar to Moss *et al*. [6], where the odor was present throughout the CDR battery).

Heuberger and Ilmberger [60] also observed that peppermint had no effect on human vigilance (for further null effects in an applied military setting see [61]). However, they reported an interaction between subjective odor ratings and task performance. That is, participants who experienced vigilance facilitation also reported an elevation in mood. This provides some evidence that any beneficial effects may have been secondary to mood changes rather than as a direct results of the pharmacological properties of the odor.

Ho and Spence [62] argue that any effect of peppermint on task performance is not an artifact of direct alertness increment *per se*, rather an increase in concentration levels under conditions of high task difficulty. In their dual-tasking study, participants concurrently performed a Rapid Serial Visual Presentation (RSVP) task (*i.e*., detection of a target digit amongst a series of distracter digits) and a vibro-tactile discrimination task (*i.e*., indicating whether vibrations were presented on the front or back of the torso). They argue that the effects of peppermint were inconsistent with what one might expect from a general alerting effect (*i.e*., faster but less accurate performance). Nor did peppermint improve RSVP (a task associated with vigilance/alertness). Instead, peppermint did enhance task performance when the response format on the vibro-tactile task was inconsistent (*i.e*., in situations when following vibrations on the front of the torso participants were required to respond by pressing the rear foot-pedal) but had no effect on reaction times. They argued that this pattern of performance is more in line with enhanced concentrations levels rather than alertness and state that peppermint facilitates the functioning of a cognitive control mechanism that inhibits inappropriate responses in the inconsistent response condition.

In contrast with peppermint, lavender (despite also being a pleasant odor) is an odor traditionally associated with soporific effects, e.g., [63]. Electrophysiological studies (EEG) have revealed that lavender exposure increased beta power [64–66]; a marker of increased drowsiness. In support of this, lavender has been found to influence activity of cyclic adenosine monophosphate [67], wherein reductions of which are associated with sedation. However, Moss *et al*. [5] reported more mixed effects in respect to self-rated and behavioral data. Following lavender presentation, self-rated measures of alertness did not significantly differ to that of the non-odor (control) condition. However, speed of attention (an aggregate measure comprising a series of reaction time measures) was significantly slower in the lavender condition relative to the non-odor control (in contrast lavender has been found to improve reaction times during a 5-min visual and auditory detection task [68], an effect purportedly due to heightened arousal). Since this effect was not mirrored in the rosemary condition, it suggests that slower responses are not an artifact of odor-induced distraction but specific to the properties of lavender [5].

Paradoxically, lavender has also been found to attenuate the deterioration of work performance under conditions of fatigue [69]. Participants were tested from 09:30–17:30 on blocks of monotonous computerized tasks. Exposure to lavender during the afternoon break attenuated the subsequent deterioration in work performance. Sakamoto *et al*. [69] argued that lavender reduced arousal levels during the recess (*i.e*., facilitated rest during the break), thereby allowing concentration levels to be higher in the following testing block.

Other odors have been shown to have specific effects on alertness/vigilance. For example, in the aforementioned Gould and Martin [10] vigilance study, bergamot was found to produce a significant detriment in performance compared to both the peppermint and non-odor conditions. Furthermore, Moss *et al*. [5] reported significantly higher alertness in the rosemary condition compared to both the lavender and non-odor groups.

In summary odor-specific effects have been observed with alertness-related paradigms, demonstrating that there is not a general effect of odors on alertness (e.g., due to division of attentional resources). The observation that pleasant odors (e.g., bergamot and lavender) can decrement alertness is further evidence against the proposition that a simple relationship exists between hedonic quality of the odor and cognition facilitation.

### 3.4. Evaluation of Mood Account

Exposure to odors can induce affective changes; however, there is insufficient evidence that mood elevation is the exclusive explanation for cognitive facilitation. Contrary to the predictions of this account, differential effects are found for odors despite being viewed as hedonically positive (indeed these differences are more pronounced if one of two positively valence odors are trigeminal, [53,70]). Furthermore, cognitive facilitation is observed in the absence of mood elevation, e.g., [6]. However, the work of Baron and colleagues [44–46] is compelling; it is entirely plausible that the induction of positive mood (via positive odors) may have a facilitative effect on cognition; however, it seems parsimonious that such benefits may be additive (rather than exclusive) to the specific effects of the odor (be that pharmacological or semantically driven).

## 4. The Role of Expectancy

In contrast to pharmacological and mood explanations, one might argue that the effects of odors on cognition are uniquely psychological in origin; that is, the expectancy of specific effects following exposure to certain odors underpins any effect rather than the chemical properties of the odor *per se* [15]. The olfactory perceptual pathway is richly interconnected with other cortical areas (including the hippocampus and amygdala) (for a review see [71]). Consequently, this interconnectivity increases the propensity for contextual and expectancy effects in odor perception. However, the expectancy hypothesis may be difficult to empirically examine since the ability to manipulate expectancy is reliant upon the participant having no prior knowledge as to the potential effects of the odor. Indeed, Howard and Hughes [72] note that a number of odor studies utilize participants with positive attitudes towards aromatherapy (although it should be noted that expectancy effects in individuals may be attenuated by their general limited ability at identifying odors, e.g., [73]). However, by using odors that are less well-known, manipulation of expectancy can enable the examination of three important ideas: (1) does presentation of the odor without expectancy provide any benefit to performance, *i.e*., are there effects due to the properties of the odor? (2) Are initial effects magnified by expectancy of those benefits? (3) Can effects be reversed through inducing expectancy that the odor will produce the opposite effects, *i.e*., can properties of the odor be overridden by expectancy?

The role of expectancy in odor-induced cognition facilitation was examined by Moss *et al*. [9], who manipulated expectations of mood changes following exposure to Roman chamomile. In a between-participants design, participants were assigned to one of the following groups: (1) receive Roman chamomile and expect arousing effects, (2) receive Roman chamomile and expect sedating effects, (3) receive Roman chamomile and given no expectancies (*i.e*., participants were deceived about the true nature of the study), and (4) no odor control. As in previous work by Moss and colleagues [5,6] participants completed the CDR battery. Moss *et al*. [9] reported that Roman chamomile does exert some effect upon cognition but that these effects are accentuated by expectancy. Specifically accuracy of attention and self-rated alertness were significantly lower and calmness was significantly higher in the no expectancy condition compared to the non-odor (control) condition. These effects suggest that Roman chamomile has sedative/relaxing properties. However, both quality of memory and secondary memory were significantly impaired in the expect sedation condition relative to the non-odor condition, illustrating that sedating effects of the odor are magnified when these effects are expected. Furthermore, these effects are reversed if participants expect the odor to have arousing properties, *i.e*., quality of memory, secondary memory, and accuracy of attention were significantly greater in the expect arousal condition compared to the expect sedation condition. These findings illustrate that, in respect to Roman chamomile, the odor does posses properties that can influence cognition. However, expectancy does influence these effects; accentuating or reversing effects depending upon prior belief (for commentary regarding the additive effects of placebo, see [74]). These findings are mirrored by Knasko *et al*. [75], who observed that individuals who (erroneously) believed that they were being presented a pleasant odor reported more pleasant mood that those who believed that they were being presented an unpleasant odor (or no odor at all); this was found despite the absence of any odor.

The above expectancy effects may be compounded in the literature by the common failure to employ an adequate placebo [72]. Specifically, comparison of a specific odor to a no-odor control (1) prevents the researcher differentiating between odor-specific effects and more general effects of odor presentation, (2) encourages demand characteristics in participants, and (3) allows experimenter expectancy effects [72]. These issues were addressed by Howard and Hughes [72] who presented participants with lavender or a non-sedative placebo odor employing an experimenter who was unaware of which odor was presented to the groups. Participants were then told that the odor would assist relaxation, inhibit relaxation, or were given no information about the odor. The authors reported no effect of odor type on physiological measures of relaxation (*i.e*., galvanic skin concordance); however, this measure was mediated by the expectancy prime (see also [76]). That is, participants demonstrated higher relaxation states if told that the odor would inhibit relaxation, purportedly because individuals tried harder to relax following the prime. The authors therefore conclude that an evaluation of participant’s knowledge and expectancies following odor exposure are an important consideration in the domain.

The above studies indicate that expectancy may provide, at the very least, an addictive influence on mood and cognition. However, the full influence of expectancy on odor-effects is unknown due to the lack of appropriate placebos and control of experimenter expectancy [72].

## 5. Semantic/Contextual Effects with Odors

The fourth proposed mechanism for odor-induced cognitive facilitation relates to odor context priming mood/behavior [15]. Rather than pharmacological properties of the odor affecting state, individuals associate a specific stimulus/behavior/mood with an odor. The close association between odors and memories has been shown both cognitively and neurologically. Engen [77] proposes that odors become intrinsically entwined with the memory, forming a holistic representation of the event. Indeed, it has been shown that odor-cued autobiographical memories contain greater detail and more emotional content than those cued by a verbal label [78], with such odor-evoked memories shown to activate the amygdala [29]. Such interconnectivity between odors, emotion, and memory reflects the close link with the limbic system during olfactory processing (for review see [71]). Furthermore, odor-specific neurons have been shown to also respond to the contextual cues associated with that odor [79–81]. Due to this interconnectivity, it is possible that representation of an odor may cue the memory, mood, or behavior present during initial odor exposure.

The pharmacological and mood accounts above are both premised on the notion that the chemical compounds of odors affect cognition either directly or indirectly following mood elevation. However, a number of studies have shown that the memorial associations made with odors are influenced by semantic knowledge rather than the pharmacological properties of the odor. For example, neural activation following odor exposure can reflect what an individual believes they are smelling rather than the actual chemical stimulus [82]. In this study participants were presented words (cheddar cheese or body odor) contemporaneously with an odor (isovaleric acid mixed with cheddar cheese flavor). When the cheese label was provided, the odor was rated as more pleasant. Furthermore, both isovaleric acid and clean air (control) showed significantly more activation in the rostral anterior cingulated cortex/medial orbitofrontal cortex when labeled as cheddar cheese. This shows that odor experience (both cognitively and neurologically) can be influenced by the top-down processing of semantic association. Consistent with these findings [82], hedonic responses to both culturally important [83–85] and novel odors have been shown to be culturally-specific [86]. Indeed, Cain [87] argued that odor discrimination is poor and that top-down processing is an essential component of the olfactory process. This further illustrates the role of experiential learning in odor perception and thus highlights how learnt associations may influence variability in odor-induced cognitive facilitation.

### 5.1. Odors Facilitating Memory via Contextual Associations

This mechanism may provide an alternative explanation for any memorial benefits reported e.g., [5], with odor exposure operating as a contextual cue to memory. Specifically, if individuals learn stimuli within a particular contextual environment, re-instatement of that environment at recall can operate as a memorial cue and facilitate recall, e.g., [88]. Indeed, Aggleton and Waskett [89] found that exposing participants to odors present at a museum, could aid recall of that experience (despite a retention interval of several years). These effects have also been shown with short-term recognition memory [90], wherein memory for incidentally acquired visual stimuli was improved if the odor presented during encoding was represented at test (see also [91]). Furthermore, Ehrlichman and Halpern [92] demonstrated that participants reported more positive memories in the presence of pleasant odors and more negative memories following exposure to unpleasant odors. One might argue, therefore, that the valence of the odor reinstated the mood context of learning and thus primed recall. Hedonic congruity between the event and the odor appears significant for such context effects. Chupnik *et al.* [93] found that odors were more accurately recognized when they had been paired to a hedonically similar narrative. Furthermore, the facilitative effects of odors in generating stories were enhanced when the mood of the odor matched that of the story. In respect to the aforementioned Moss *et al*. [5,6] findings, participants were exposed to rosemary and peppermint, respectively, during both learning and recall. Since odor contexts were consistent at learning and recall, these odor contexts may have facilitated recall.

A clear limitation for this contextual explanation is the observation that memorial facilitation was not found for lavender [5] or ylang-ylang [6]. In both studies the odor context was consistent for learning and recall, therefore an odor contextual cue was present at recall. These anomalous context effects were examined by Ball *et al*. [94] who used a fragment word completion task to examine the effects of learning and recalling in same odor contexts. In Experiment 1 participants were assigned to one of the following groups: (1) learning with lemon, recalling with lemon, (2) learning with lemon, recalling with rosemary, (3) learning with rosemary, recalling with rosemary, and (4) learning with rosemary, recalling with lemon. They [94] reported a context effect, however, this effect was only apparent for rosemary, *i.e*., a recall advantage was found for participants who learned and recalled with rosemary but not for participants who learned and recalled with lemon. They [94] argued that such an effect is driven by both unpleasantness and distinctiveness, wherein the greater the unpleasantness/distinctiveness of the odor, the more salient the context. This was broadly supported in Experiment 2 where unpleasant/distinctive (rosemary), unpleasant/non-distinctive (hyssop), pleasant-distinctive (lemon), and pleasant/non-distinctive (orange) odors were compared where participants learned and recalled in the same odor context. Distinctiveness and unpleasantness were found to have additive effects on recall, *i.e*., the strongest context effects were found with the distinctive/unpleasant odor (*i.e*., rosemary). These findings may explain why some memorial benefits were found with rosemary and not lavender [5], since the unpleasant/distinctive characteristics of rosemary produced a sufficiently salient context to induce a context dependent memory effect but lavender did not. However, since Ball *et al.* [94] did not employ a control (no odor) condition; it is unclear whether the memorial benefits reported were uniquely underpinned via contextual re-instatement or additive following general odor-induced memorial facilitation.

### 5.2. Odors Affecting Mood via Contextual Associations

Affective responses to odors have been shown to influence cognition [44]. However, these affective responses may be underpinned via contextual/semantic associations with the odors rather than the pharmacological properties of the odor *per se*. As described above, both semantic information [82] and cultural experience [83–85] can determine hedonic evaluation of an odor. However, mere contiguous presentation of an odor alongside a mood can result in future reinstatement of the odor cueing the associated mood, e.g., [95]. For example, presentation of a novel ambient odor during performance of a frustration-inducing task resulted in the formation of an association between the two stimuli, wherein subsequent re-presentation of that odor during an unrelated task resulted in reduced motivation and inferior performance [96,97].

### 5.3. Odors Affecting Decision Making via Contextual Associations

Odor concepts are framed within a semantic context and presentation of the odor has been shown to cue behavior related to that semantic context. For example, in a lexical decision making task, participants were faster at responding to cleaning-related words if the task was conducted in the presence of a cleaning scent [98]. More profoundly, participants were more likely to plan to clean later in the day and even engage in cleaning type behaviors during the experiment if a cleaning scent was presented [98]. In such a situation Holland *et al*. [98] suggest that “behavior is brought in line with semantic associations that become activated upon the perception of a scent” (p. 692). In a further study, consumer decision making was found to the qualitatively altered by the type of odor presented [14]. If the odor was congruent with the product, then the decision making process was found to be more in-depth, with incongruent odors argued to cause interference in decision making [14]. Both studies illustrate how the semantic/contextual association with an odor can both quantitatively and qualitatively alter decisions.

### 5.4. Evaluation of Semantic Account

It is clear that the semantic associations can influence the perception of odors [82]. In addition, perception of that odor can trigger semantically associated cognitions and behaviors [98]. The strong associations between memories and odors [91] may provide a mechanism for why certain odors may cue cognitive facilitation. However, the effects of ingested essential oils (e.g., [22]) coupled with ability to disrupt the semantic association via an expectancy manipulation (e.g., [9]), suggests that semantic/contextual associations are not the unique mechanism for cognitive facilitation. Indeed, analogous to the role of expectancy, one might argue that contextual effects are additive rather than uniquely responsible for observed improvements.

## 6. Conclusions

This review has shown that odors arising from urban pollution are not the only circumstances in which odors may impact cognition. The intentional utilization of odors in both commercial/industrial and private settings can have a direct impact upon cognition which is not exclusively beneficial. As a consequence, decisions to employ such odors needs to be empirically-driven or such interventions may have a detrimental effect on performance. Commercially available odors have been shown to facilitate and decrement both measures of memory and alertness. Effects are, therefore, not simply related to the hedonic quality of the odor.

Evidence for the four potential explanations of odor facilitation [15] has been reviewed. Essential oil compounds internalized without inhalation can have a direct influence on cognition; however, this does not necessarily show that the pharmacological properties of the odor induce post-inhalation effects. Indeed, more work is required to find evidence of olfactory compounds entering the human system following inhalation. It is though apparent that odor effects can be accentuated/attenuated via improvements to mood, expectancy of certain effects, and contextual congruity. More research is required in respect to each of these accounts. It needs to be established whether the chemical compounds within odors can facilitate/decrement specific cognitive operations; if this is shown, those compounds require isolation. Indeed, a more refined summary of the specific effects of a range of odors would enable the appropriate employment of certain odors to certain circumstances (*i.e*., matching odors to specific tasks/requirements). Furthermore, it is unclear how quickly individuals habituate to such benefits; data is required on the longevity of such effects in order to rationalize the financial outlay. In respect to the mood elevation hypothesis, it is important that mood measures are taken in conjunction with cognitive measures. Such an assessment would enable evaluation of the situations in which cognition improves independently of mood elevation. Limited research has been conducted on the role of expectancy, however initial data indicate that expectancy can accentuate/attenuate the effects of an odor [9,71]. Further research is required to ascertain the tasks that can be influenced via expectancy of odor-related effects and whether such expectancy effects are robust over prolonged odor exposure. Finally, odors can be used as contextual cues to cognitions, behavior, and mood. It is, however, unclear the extent to which odors can be used to cue behaviors/moods across a range of associations and whether the formation of such associations are odor-specific.
